# Potential drug-drug interactions in drug therapy for older adults with chronic coronary syndrome at hospital discharge: A real-world study

**DOI:** 10.3389/fphar.2022.946415

**Published:** 2022-08-24

**Authors:** Mei Zhao, Chuan-Fen Liu, Yu-Fei Feng, Hong Chen

**Affiliations:** ^1^ Department of Pharmacy, Peking University People’s Hospital, Beijing, China; ^2^ Department of Cardiology, Peking University People’s Hospital, Beijing, China; ^3^ Beijing Key Laboratory of Early Prediction and Intervention of Acute Myocardial Infarction, Peking University People’s Hospital, Beijing, China; ^4^ Center for Cardiovascular Translational Research, Peking University People’s Hospital, Beijing, China

**Keywords:** drug-drug interactions, chronic coronary syndrome, older adults, discharge, drug therapy

## Abstract

**Introduction:** Polypharmacy are commonly observed among older adults with cardiovascular disease. However, multiple medications lead to increased risk of drug-drug interactions (DDIs). Therefore, identification and prevention actions related to harmful DDIs are expected in older adults. The study aimed to describe the prevalence of potential DDIs (pDDIs) in discharge prescriptions among older adults with chronic coronary syndrome (CCS).

**Methods:** A single-center cross-sectional study was performed in a tertiary public hospital in Beijing, China. CCS patients aged 65 years and above who were admitted to cardiology wards over a 3-month period and alive at discharge were included. Electronic medical records and discharge prescriptions were reviewed. pDDIs were evaluated through the Lexi-Interact online.

**Results:** pDDIs were identified in 72.9% of the 402 individuals (*n* = 293). A total of 864 pDDIs were obtained. 72.1% of patients were found with C DDIs (*n* = 290) and 20.3% were categorized in D and X DDIs (*n* = 82). The only X DDI was between cyclosporine and atorvastatin. Under category D, glycemia alterations within antidiabetics and increased chances of bleeding with antithrombotic were the most common. Concomitant use of clopidogrel and calcium channel blockers was a frequent situation within category C, followed by synergic blood pressure lowering agents and increased rosuvastatin concentration induced by clopidogrel.

**Conclusion:** DDIs exposure was common in older CCS. DDIs screening tools should be introduced to alert potential adverse effects. Prescribers need to rigorously review or modulate therapies to prevent DDI-related adverse outcomes. Clinical pharmacists should be more involved in complex drug regimen management.

## Introduction

Drug-drug interactions (DDIs) are defined as alterations in effectiveness or toxicity when drugs are co-administered (Hines and Murphy, 2011). DDIs pose significant challenges in adverse drug events (ADEs), hospital admissions, rehospitalization and emergency visits (Becker et al., 2007; Magro et al., 2012; Gatenby et al., 2020; Limandri, 2020). Concomitantly, this results in increased hospital stays and health care costs (Thomsen et al., 2007; Moura et al., 2009). Therefore, DDIs management is crucial for the improvement of medication safety.

The group with a high risk of DDIs was defined as advanced age, a diagnosed cardiovascular system disorder, complex medication regimen and so on (Yoon et al., 2018; Gallo et al., 2019; Veloso et al., 2019). Given that polypharmacy was commonly observed for the treatment of concurrent chronic conditions, it can be expected that the prevalence of DDIs among older adults will inherently increase (Prince et al., 2015; Yoon et al., 2018; Lea et al., 2019; Ruangritchankul et al., 2020). Notably, older adults were also reported an identifiable a high degree of DDIs in risk rating (e.g., major or severe). For example, 60% older cancer adults in French and 21% of geriatric cases in India were suffering from major DDIs (Nightingale et al., 2018; Shetty et al., 2018). The main reason is that decreased physiological reserves with age results in pharmacokinetic and pharmacodynamic alterations. Conceivably, pervasive use of medications combined with elevated vulnerability to drug effects will exacerbate the likelihood of DDIs exposure (Beinse et al., 2020).

Coronary artery disease (CAD) remains an emerging threat for older people among COVID-19 pandemic (Prince et al., 2015; Zhao et al., 2019; Hessami et al., 2021). Evidence-based medication therapy is emphasized as sacrosanct and lifelong (Bansilal et al., 2015; Knuuti et al., 2020). At the same time, increased medication use has developed a substantial proportion of drug-related problems, including DDIs, ADEs and poor adherence (Gelchu and Abdela, 2019; Plácido et al., 2020; Tsige et al., 2021). A study done in Ethiopia showed that 47.0% of heart failure were exposed to severe DDIs, which were the most common drug therapy problems (Seid et al., 2020). Chronic coronary syndrome (CCS) is a broad group of CAD proposed by the European Society of Cardiology (Knuuti et al., 2020; Ferrari et al., 2021). The presence of CCS nearly doubles the risk of major adverse cardiovascular events (Romero-Farina and Aguadé-Bruix, 2021). All the current literatures advocate the timely medical therapy for CCS patients (Yasuda et al., 2018; Silber, 2019; Zahmatkeshan et al., 2021). Consequently, multiple drug use as well as potential DDIs (pDDIs) are anticipated in older CCS adults. Our previous findings revealed that DDIs accounted for 30% of potentially inappropriate medications in older CCS (Zhao et al., 2021). Unfortunately, fewer studies properly examine pDDIs among older CCS patients in China. As a results, insight into pDDIs is a huge opportunity for clinicians to predict and avoid ADEs and reduce hospital readmission.

In this regard, the aim of the present study was to quantify the prevalence of pDDIs among a group of older patients with CCS from real-world data and to analyze the most common pDDIs in discharge prescriptions.

## Materials and methods

### Study design and setting

A cross-sectional study was carried out in Peking University People’s Hospital, a major public tertiary teaching center in Beijing, China. This study was approved by the Ethics Committee of Peking University People’s Hospital and was granted an exemption of informed consent from patients. The information was collected from the electronic medical records anonymously and used for research only.

A sample size of 387 patients was calculated regarding the prevalence of DDIs as 60% (Fettah et al., 2018), with a two-sided 95% confidence interval with a width equal to 0.10.

### Participants

Older adults (aged over 65 years) with CCS who were admitted to the cardiology department between October and December 2020 and alive at discharge were included in this study. Only patients with two or more medications at discharge were selected for this investigation.

### Data collection and software used for potential drug-drug interactions identification

Demographic and clinical information, including age, sex, diagnosis, the New York Heart Association (NYHA) class and comorbidities was obtained.

Medication regimens often changed during hospitalization. Hospital discharge prescriptions pose patients at new risks of ADEs (Alqenae et al., 2020; Grandchamp et al., 2022). Usually, upon discharge, the attending physician would prescribe a comprehensive discharge prescription based on the patient’s diagnosis. Therefore, prescriptions at discharge were collected through the electronic medical records. The Anatomic-Therapeutic-Chemical (ATC) Drug Classification (20th Ed., 2017) formulated by the World Health Organization Collaborating Centre was used for drug classification.

The medication regimens for pDDIs were analyzed using the Lexi-Interact online (Lexi-Comp Inc., Hudson, United States). As a computerized software, easy access to Lexi-Interact is recognized as a benefit. Lexi-Interact succinctly provides information about the risk, reliability and severity of pDDIs. It also elaborates recommendations on the prevention and management of pDDIs. This database classifies pDDIs into five risk rating according to the degree of clinical significance (category A, B, C, D, and X). In most of studies, C, D and X were considered potential clinically relevant DDIs. Depending on the quality of evidence, reliability is classified as excellent, good and fair-type. Severity indicators include major, moderate and minor. Table 1 lists the definitions of the risk rating, reliability rating and severity rating by the Lexi-Interact database (Moradi et al., 2020). For the purpose of this study, the category C, D and X, reliability rating and severity rating were searched. Clinical consequences and management strategies also conformed to the Lexi-Interact monograph.

**TABLE 1:** Definitions of risk, reliability and severity ratings for DDIs by Lexi-Interact software.

Classification	Definition
risk rating	The level of urgency and actions needed to respond to DDIs
A	No known interaction
B	No action needed
C	Monitor therapy
D	Consider therapy modification
X	Avoid combination
reliability rating	The quantity and nature of evidence
excellent	Multiple clinical trials or single clinical trial plus more than two case reports
good	Single randomized clinical trial plus less than two case reports
fair	More than two case reports or less than two case report plus other supporting data; or a theoretical interaction based on known pharmacology
severity rating	Qualify the reported or possible magnitude of DDIs outcomes
major	The effects of DDIs might be life-threatening or cause permanent damage
moderate	Patients with DDIs may require additional care
minor	The effects of DDIs may be tolerable and need no medical interventions

DDIs, drug-drug interactions.

### Statistical analysis

Statistical analysis was performed using IBM SPSS Statistics for Windows, version 23.0 (IBM Corp, Armonk, NY, United States). Categorical data are presented as frequencies or percentages, and continuous data are presented as the mean ± SD or median and interquartile range (IQR).

## Results

### Main characteristics of older chronic coronary syndrome patients

402 eligible older CCS patients who met the inclusion criteria received at least two dispensing at discharge. Overall, females made up 41.8% of the total population. The mean age was 73.8 ± 6.3 years (range 65–90). The NYHA classification of the patients was as follows: 55.7% in NYHA I, 31.1% in NYHA II, and 13.2% in NYHA III and IV. The median number of comorbidities was 5 (range 0–13); hypertension was prominent (77.1%), followed by dyslipidemia (65.7%), peripheral arterial disease (53.5%) and type 2 diabetes mellitus (42.3%). The median length of the hospital stay was 7 days (range 1–33). The general characteristics of the 402 patients are described in Table 2.

**TABLE 2:** Characteristics of the study sample (N = 402).

Characteristics	n (%)
Sex	
Male	234 (58.2)
Female	168 (41.8)
Age (years)	
Mean ± SD	73.8 ± 6.3
Length of stay (days)	
Median, IQR	7 (5–9)
NYHA class	
I	224 (55.7)
II	125 (31.1)
III	43 (10.7)
IV	10 (2.5)
Number of comorbidities	
Median, IQR	5 (3–6)
Cardiovascular comorbidities	
Hypertension	310 (77.1)
Dyslipidemia	262 (65.2)
Peripheral arterial disease	215 (53.5)
Type 2 diabetes mellitus	170 (42.3)
Stroke	90 (22.4)
Atrial fibrillation	70 (17.4)
Heart failure	50 (12.4)
Non-cardiovascular comorbidities	
Tumor	55 (13.7)
Chronic kidney disease	54 (13.4)
Psychiatric disorders	38 (9.5)
Benign prostatic hyperplasia	36 (9.0)
Thyroid dysfunction	35 (8.7)
GERD/peptic ulcer	34 (8.5)
COPD/asthma	24 (6.0)
Chronic liver disease	10 (2.5)

COPD, chronic obstructive pulmonary disease; GERD, gastroesophageal reflux disease; IQR, interquartile range; NYHA, New York Heart Association.

### Prevalence and characteristics of potential drug-drug interactions in discharge prescriptions

**FIGURE 1:**
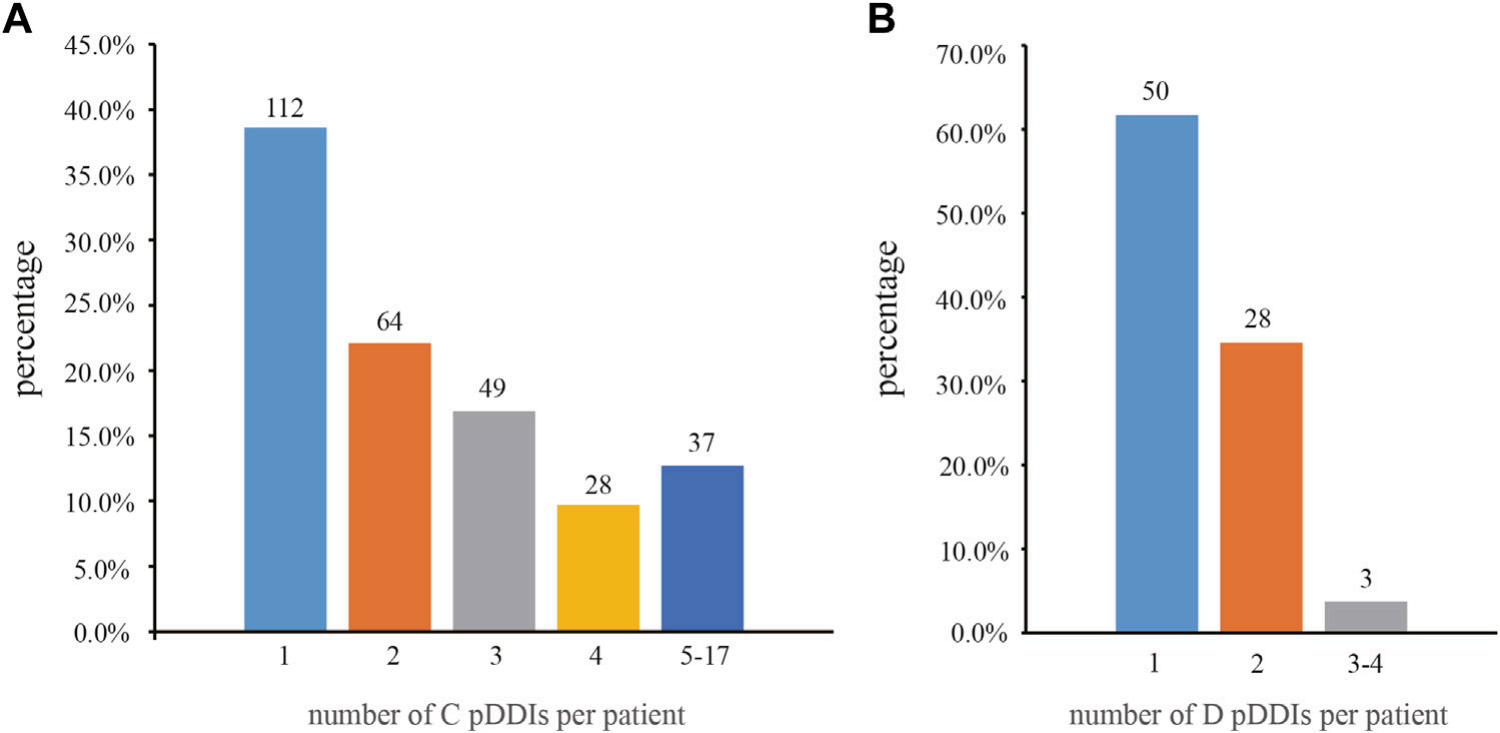
Frequency and percentage of pDDIs per patient based on risk category. **(A)** category C (*n* = 290); and **(B)** category D (*n* = 81). Percentage was calculated out of number of patients with C or D pDDIs. pDDIs, potential drug-drug interactions.

A total of 2,669 medications were prescribed at discharge, with an average of 6.6 ± 2.2 per patient. pDDIs were found in 293 patients (72.9%) with 864 pDDIs in all (Table 3). 202 patients were observed within three pDDIs (50.2%), while six individuals (1.5%) showed more than ten simultaneous pDDIs. The median number of pDDIs was 2 (range 1–17). With regard to the risk category, the vast majority of patients were exposed to class C (*n* = 290, 72.1%), followed by class D (*n* = 81, 20.1%) and class X (*n* = 1, 0.2%). Figure 1 showed the distribution of pDDIs per patient based on risk category. Thirty seven individuals had the most distribution of 5–15 category C pDDIs, and only three patients had 3, 4 category D pDDIs.

**TABLE 3:** Prevalence of pDDIs among older CCS patients at discharge.

Characteristics	Patient, n (%)
Total number of medications	2,669
Mean prescribed drugs per patients	6.6 ± 2.2
Patients with pDDIs^a^	293 (72.9)
Number of pDDIs per patient^a^	
1	99 (24.6)
2	62 (15.4)
3	41 (10.2)
4	30 (7.5)
5	25 (6.2)
6–9	30 (7.5)
10–17	6 (1.5)
Total number of pDDIs	864
Median (IQR) of pDDIs per patient	2 (1–4)
Patient distribution based on risk category^a^	
C	290 (72.1)
D	81 (20.1)
X	1 (0.2)

a Percentage was calculated out of the total number of CCS patients (*n* = 402).

CCS, chronic coronary syndrome; pDDIs, potential drug-drug interactions; IQR, interquartile range.

Out of 864 drug pairs we considered, 747 fell under category C (86.5%), 116 fell under category D (13.4%) and one fell under category X (0.1%). In terms of reliability, 22 (2.5%) pDDIs were excellent, 246 (28.5%) pDDIs were good, and 596 (69.0%) were fair-type. According to the Lexi-Interact classification, severity was mainly attributed to moderate (760 pDDIs, 87.9%) and major (87 pDDIs, 10.1%) (Table 4).

**TABLE 4:** Characteristics of drug interactions at discharge.

Characteristics	n (%)^a^
Risk rating	
C	747 (86.5)
D	116 (13.4)
X	1 (0.1)
Reliability rating	
Excellent	22 (2.5)
Good	246 (28.5)
Fair	596 (69.0)
Severity rating	
Major	87 (10.1)
Moderate	760 (87.9)
Minor	17 (2.0)

a %: percentage was calculated out of the total number of pDDIs (*n* = 864).

### Drug classes involved in potential drug-drug interactions

**FIGURE 2:**
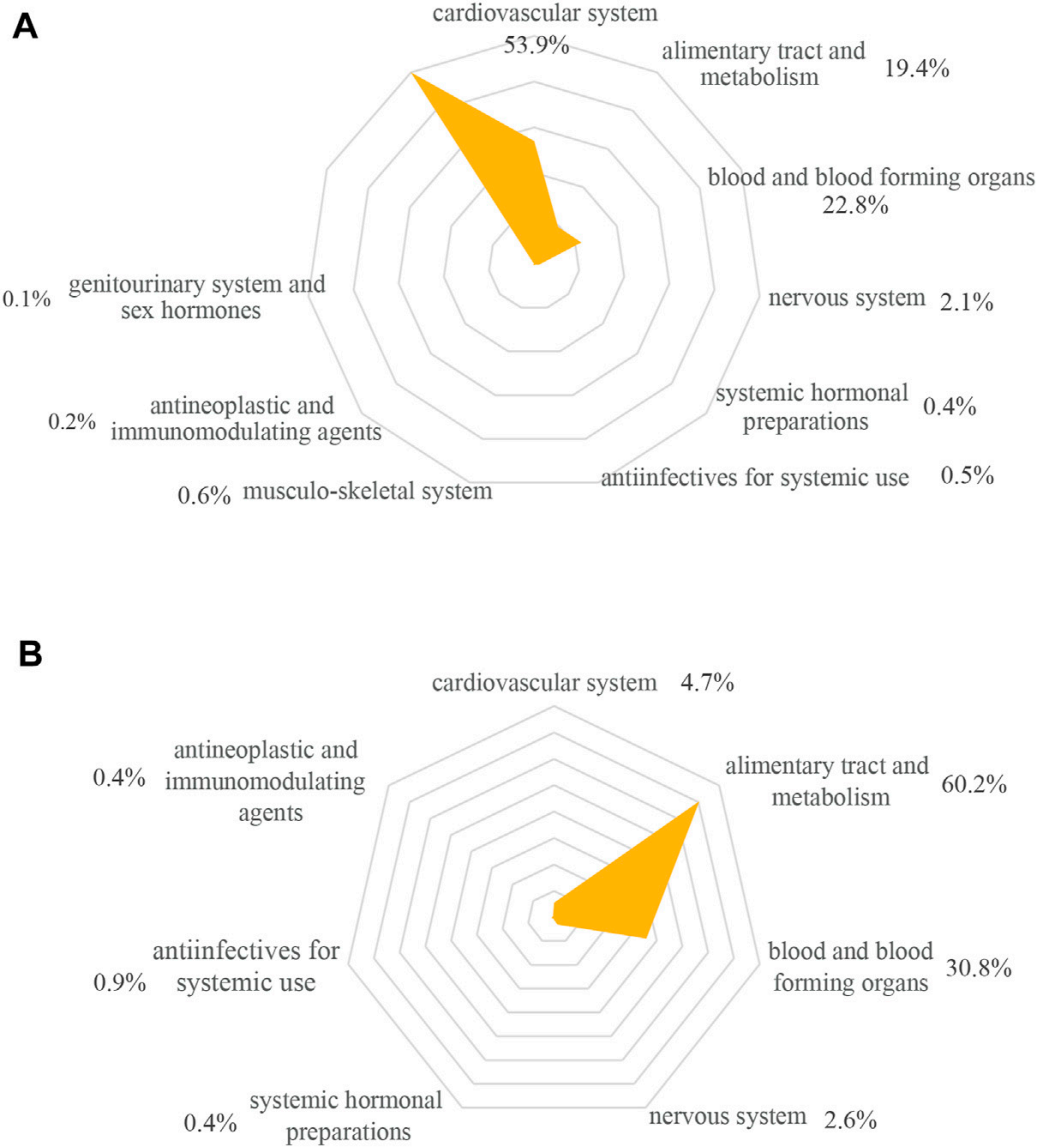
ATC classification-wise distribution of pDDIs. **(A)** category C (*n* = 1,494); **(B)** category D and X (*n* = 234). Percentage was calculated out of number of pDDIs in each risk category. pDDIs, potential drug-drug interactions.

In general, nine ATC groups were involved in category C pDDIs (Figure 2). The significantly associated drug class was drugs related to the cardiovascular system (53.9%, 806/1494). Then followed by blood and blood forming organs (22.8%, 340/1494) and the alimentary tract and metabolism (19.4%, 291/1494). Among the seven ATC groups relevant to category D and X, alimentary conditions and metabolism classification increased the exposure to DDIs (60.2%, 141/234) (Figure 2).

Supplementary Table S1 presented the ATC classification of drugs. Regarding category C pDDIs, the highest frequency was found in antiplatelets (331), diabetes drugs (266), calcium channel blockers (CCBs, 179) and diuretics (176). The highest prevalence of interacting drugs within category D and X were attributed to antidiabetics (130), followed by antiplatelets (41) and anticoagulants (26) (Supplementary Table S1).

### The most frequently observed drug pairs

Table 5 described the most frequently observed drug pairs and potential adverse effects. The exclusive contraindicated pair was between cyclosporin and atorvastatin. A dominant potential outcome of category D was hypoglycemia related to synergistic hypoglycemic action and the concurrent use of repaglinide with clopidogrel (69, 59.4%). Then it was followed by agents that elevated the risk of bleeding (29, 25.0%).

**TABLE 5:** Most frequently occurring DDIs and management strategies.

Drug pairs	n (%)^a^	Potential consequence	Management strategies
Category X	1		
Cyclosporine + atorvastatin	1 (100.0)	Myopathy	Change to pravastatin or fluvastatin or an alternative type of LDL-lowering medication
Category D	116		
Glycemia alterations	69 (59.4)		
Antidiabetic drugs (e.g. insulin/sulfonylurea with acarbose/sitagliptin/SGLT2 inhibitor/thiazolidinedione)	61	Hypoglycemia	Monitor glucose; a decrease in insulin/sulfonylurea dose
Clopidogrel + repaglinide	8	Hypoglycemia	Monitor glucose; titrate repaglinide with a limit of 4 mg daily
Additive bleeding risk	29 (25.0)		
Antiplatelets + oral anticoagulants	27	Bleeding	Monitor signs of bleeding
Warfarin + amiodarone	2	Bleeding	Monitor INR; warfarin dosage reduction
Omeprazole/fluconazole + clopidogrel	6 (5.2)	Decreased antiplatelet effect of clopidogrel	Replacement with rabeprazole or pantoprazole or alternatives of azole
Amlodipine + simvastatin	3 (2.6)	Muscle toxicity	Monitor signs of myopathy; limit simvastatin to 20 mg daily
QT prolongation or serious arrhythmias	3 (2.6)	Serious arrhythmias or death	Monitor ECG
Sodium bicarbonate + polysaccharide-iron complex	2 (1.7)	Reduced effect of iron preparations	Separate oral administration moments
Potassium chloride + spironolactone	2 (1.7)	Hyperkalemia	Monitor potassium concentration
Calcium carbonate + levothyroxine	1 (0.9)	Reduced levothyroxine effect	Separate at least 4 h
Quetiapine + levodopa	1 (0.9)	Diminished levodopa effect	A non-dopamine antagonist alternative
Category C	747		
CCBs + clopidogrel	110 (14.7)	Reduced antiplatelet effect	Monitor platelet reactivity index
Blood pressure lowering drugs (e.g., sacubitril/valsartan, renin-angiotensin system inhibitors, β blocking agents, diuretics and CCBs)	97 (13.0)	Enhanced hypotensive effects	Monitor blood pressure
Clopidogrel + rosuvastatin	93 (12.4)	Myopathy	Monitor the signs of myopathy and liver function test
Diuretics + antidiabetic agents	71 (9.5)	Reduced antidiabetic effect	Monitor blood glucose
*β* blockers + insulin/sulfonylureas	63 (8.4)	Mask hypoglycemia	Monitor blood glucose
Hypoglycemic agents combination (e.g., metformin, repaglinide, sulfonylureas, insulin)	41 (5.5)	Hypoglycemic effect	Monitor blood glucose
Aspirin + diuretics (e.g., loop diuretics and spironolactone)	38 (5.1)	Nephrotoxicity and diminished diuretics effects	Monitor serum creatinine and diuretic response
Aspirin + ACE inhibitors	32 (4.3)	Nephrotoxicity	Monitor renal function

a %: percentage was calculated out of the number of pDDIs in each risk category.

ACE, angiotensin converting enzyme; CCB, calcium channel blocker; CYP, cytochrome; LDL, low density lipoprotein; OATP, organic anion transporting polypeptide; PD, pharmacodynamics; p-gp, p-glycoprotein; PK, pharmacokinetics; SGLT, sodium-glucose cotransporter.

Exposure to clopidogrel and CCBs (110, 14.7%), as assigned to one main class C interaction, might lead to a reduced antiplatelet response with clopidogrel. Then there were drug interactions that affected blood pressure and lipids (97, 13.0% and 93, 12.4%, respectively). Notably, glycemia fluctuation was more visibly seen in diabetes who used diuretics or *β* blockers simultaneously (134, 17.9%). Moreover, in the aspirin group, loop diuretics, spironolactone and angiotensin converting enzyme inhibitors had an enhanced possibility of renal dysfunction (70, 9.4%).

### Management strategies

The Lexi-Interact monograph also provides skilled DDIs management, as shown in Table 5. Adjustment in treatment regimens was required in category X and most category D pDDIs. Adjustments included dosage reduction, e.g. insulin, sulfonylurea and warfarin, titration e.g., repaglinide with a limit of 4 mg daily and simvastatin to 20 mg daily, separate administration time and drug replacement. Vigilant signs/symptoms and lab tests were widely recommended in class C pDDIs, including platelet reactivity index, blood pressure, blood glucose, liver/renal function and any signs or symptoms of myopathy.

## Discussion

Polypharmacy is a major concern for older individuals (Soejono and Rizka, 2021). Multiple drugs carries a high risk of DDIs, and their associated adverse events vary from minor toxicity to treatment failure or even death (Malki and Pearson, 2020; Davies and O'Mahony, 2015). Our present study revealed that a high proportion of older CCS patients were exposed to pDDIs; furthermore, one fifth were classified as severe and contradictory pDDIs. pDDIs mostly involved drugs acting on the cardiovascular system, alimentary tract and metabolism, and blood and blood forming organs. It is very crucial for healthcare providers to have this data and help manage drug usage for better scheduling and planning.

Overall, the prevalence of pDDIs in CCS was higher than that in certain other scenarios, such as cancer (18.7%), intensive care unit stays (54%), dementia (43.2%), liver cirrhosis (21.5%) and COVID-19 (38%) (Franz et al., 2012; Uijtendaal et al., 2014; Sönnerstam et al., 2018; Vecchia et al., 2018; Mahboobipour and Baniasadi, 2021). Our findings were comparable with previous studies of DDI prevalence in non-acute cardiac inpatients, such as 100% in Pakistan, 61% in Serbia and 68% in Morocco (Fettah et al., 2018; Kovačević et al., 2020; Akbar et al., 2021). Medication complexity could partly explain the sizable DDIs (Forman et al., 2018). For example, all patients with acute coronary syndrome were experiencing pDDIs with 9.4 drugs on average, while only 33.4% in hypertension with daily drug use as 4.3 (Pejčić et al., 2019; Ersoy and Ersoy, 2021). Discrepancy in pDDIs could also be due to using different screening tools. In comparison of five DDI programs, including Lexi-Interact, Micromedex, iFacts, Medscape and Epocrates, Lexi-Interact and Micromedex showed the best performance on accuracy and sensitivity (Kheshti et al., 2016). Lexi-Interact was widely used in various diseases and different areas (Ren et al., 2020; Dagdelen et al., 2021; Ramsdale et al., 2022). Meanwhile, Lexi-Interact was available in our health system, as such, pDDIs were reviewed using Lexi-Interact software in this study.

In our study, DDIs of clinical significance were most frequently observed in category C. Pharmacokinetic drug interactions affect at the steps of absorption, distribution, metabolism and elimination. It has been established that the inhibition of CYP3A4 by dihydropyridine CCBs and the inhibition of P-glycoprotein by several CCBs (diltiazem, verapamil and nifedipine) were potentially harmful in clopidogrel biotransformation (Gremmel et al., 2015). However, controversy persisted as to whether CCBs modified the clinical protection of clopidogrel and subsequent changes in major adverse cardiovascular end points (Good et al., 2012; Aggarwal et al., 2016). Until now, it is difficult to determine clopidogrel resistance resulting from the co-administration of CCBs. Monitoring genetic polymorphisms or switching to ticagrelor or prasugrel might be considered for those with low efficacy of clopidogrel (Wang et al., 2015).

Most patients with hypertension required multiple drugs, such as sacubitril/valsartan or rennin-angiotensin system inhibitors with diuretics, *β* blockers or CCBs (Ersoy and Ersoy, 2021). However, pharmacodynamic DDIs lead to synergic blood pressure lowering, and can reduce cerebral perfusion, presenting as syncope or falls. Older adults who are taking diuretics and polypharmacy is projected a higher incidence of falls (Abu et al., 2021). Physicians and pharmacists may need to conduct a thorough assessment of antihypertensive medications as well as hidden antihypertensive medications, such as tamsulosin and levodopa (Alagiakrishnan, 2015). It is critical to emphasize blood pressure monitoring and gradual titration to a tolerance (Oliveros et al., 2020).

Nowadays, combined use of clopidogrel and rosuvastatin is common in practice. However, Pinheiro et al. (2012) reported that clopidogrel introduced impressive growth in the AUC of rosuvastatin. Meanwhile, abnormal liver function could be found in chronic heart failure (Tavazzi et al., 2008). Inhibition of intestinal breast cancer resistance protein (BCRP) transporters by clopidogrel is likely to be a contributor of hepatotoxicity (Ning et al., 2021). Once daily clopidogrel is advised to be taken either in the morning or evening, while rosuvastatin in the evening.

Two-fifths of CCS patients in this study had type 2 diabetes mellitus. Meta analysis showed thiazide diuretics and β blockers increased the risk of developing new-onset diabetes (Nazarzadeh et al., 2021). The diuretic-decreased pharmacologic response was related to a reduction in insulin secretion secondary to potassium loss. The mechanism of β-blocking agents on glycemia-related adverse events is complex, including increased insulin resistance and the inhibition of adrenergic-mediated insulin release (Jain et al., 2017). Carvedilol seemed superior to metoprolol with a lower impact on glycemic control and more benefits on metabolic syndrome (Bakris et al., 2004). It is necessary to monitor blood glucose and refine the selection of drug choice according to an individual’s risk/benefit profile.

Rhabdomyolysis particularly occurs with drugs that potentiate statin concentration. The only interaction of category X was cyclosporine-atorvastatin regimen. Cyclosporine acts as an inhibitor of CYP3A4, p-gp and OATP1B1, resulting in a drastically elevated atorvastatin level (Bellosta and Corsini, 2018). Fluvastatin or pravastatin might be prudent to choose for CCS patients already treated with cyclosporine (Horodinschi et al., 2019).

For decades, emergency department visits for ADEs in older adults were primarily concerned with the augmented proportion of anticoagulants, antiplatelets and antidiabetics (Shehab et al., 2016). In line with this, category D DDIs at large were noted to cause detrimental hypoglycemia and bleeding. To date, add-on therapy was more prevalent than metformin monotherapy in older patients (Kim et al., 2019). Nevertheless, glucose-lowering agents might be associated with serious hypoglycemia when used in conjunction with sulfonylureas or insulin (Gómez-Huelgas et al., 2020). Both SGLT2 inhibitors and glucagon-like peptide-1 receptor agonists (GLP-1 RA) have been proven to reduce major adverse cardiovascular events with little risk of hypoglycemia (Bertoccini and Baroni, 2021). The utilization of both drugs in the present study was at a low frequency (2.4% for SGLT2 inhibitors and 5.3% for GLP-1 RA). Mitigation of hypoglycemia risk could be achieved by the selection of appropriate antidiabetic drugs, glucose self-monitoring and education on hypoglycemia symptoms.

Another challenge was to maintain balance with regards to ischemic and bleeding risks in CCS with atrial fibrillation. Co-prescription of anticoagulants with antiplatelets, especially in triple therapy, increased the absolute risk of bleeding (Michniewicz et al., 2018). Meta-analysis supported novel oral anticoagulants plus a P2Y12 inhibitor in atrial fibrillation experiencing post-percutaneous coronary interventions (Lopes et al., 2020). Good clinical judgment on drugs with better efficacy, dosage and duration is vital in patients management.

pDDIs is prevalent in older CCS patients, indicating a need to evaluate medication safety and strict monitoring during CCS treatment. DDI screening and alerting systems should be implemented in electronic medical records (Celebi et al., 2019; Horn and Ueng, 2019; Anrys et al., 2021). Pharmacist-driven prescription review system in real time has been allowed to optimize therapy (Lineberry et al., 2021). In certain instances, a multidisciplinary team with a physician, a pharmacist and a nurse was required especially in complex drug regimens (Silva et al., 2015; Aghili and Kasturirangan, 2021). Clinical pharmacists should also make attempts at patient education and counseling to reduce the incidence of serious or fatal DDIs (Riu-Viladoms et al., 2019).

The results of the current real-life setting yields pragmatic information on medications that might pose risk in older CCS patients. Some limitations should be considered. The current design focused on pDDIs and did not identify actual clinical manifestations, such as persistent use and doses of drugs. A follow-up for potential clinical outcomes and relevant interventions is required. Second, a multicenter study might allow data to be more generalizable. Third, although the wide use of Lexi-Interact database, it could not provide information on whether drug combinations were appropriate in certain circumstances. For instance, valsartan and potassium chloride are sometimes concomitantly used in an implantable cardioverter-defibrillator recipient with hypokalemia. Fourth, older adults in China preferred to take herbs as self-medications, and many of them were unwilling to inform doctors or clinical pharmacists. As a result, potential interactions between medicines and herbs tend to be underestimated.

## Conclusion

The present study showed a substantial proportion of older CCS patients were exposed to pDDIs at discharge, and one fifth were involved in serious or contraindicated DDIs. Thus, judicious clinicians should be more knowledgeable and cautious in recognizing and minimizing undesirable adverse events. In the multidisciplinary team, well-trained clinical pharmacists are responsible for comprehensive medication reviews. Furthermore, data obtained in this study can be used to design DDIs screening and alert interventions to optimize patient care.

## Data Availability

The original contributions presented in the study are included in the article/Supplementary Material, further inquiries can be directed to the corresponding author.

## References

[B1] Abu B. A. Abdul K. A. Idris N. S. Mohd N. S. (2021). Older adults with hypertension: Prevalence of falls and their associated factors. Int. J. Environ. Res. Public Health 18 (16), 8257. 10.3390/ijerph18168257 34444005 PMC8392439

[B2] Aggarwal S. Loomba R. S. Arora R. R. (2016). Effects of concurrent calcium channel blocker on antiplatelet efficacy of clopidogrel therapy: A systematic review. Am. J. Ther. 23 (1), e29–36. 10.1097/MJT.0000000000000225 26745332

[B3] Aghili M. Kasturirangan M. N. (2021). Management of drug-drug interactions among critically ill patients with chronic kidney disease: Impact of clinical pharmacist's interventions. Indian J. Crit. Care Med. 25 (11), 1226–1231. 10.5005/jp-journals-10071-23919 34866818 PMC8608633

[B4] Akbar Z. Rehman S. Khan A. Khan A. Atif M. Ahmad N. (2021). Potential drug-drug interactions in patients with cardiovascular diseases: Findings from a prospective observational study. J. Pharm. Policy Pract. 14 (1), 63. 10.1186/s40545-021-00348-1 34311787 PMC8311960

[B5] Alagiakrishnan K. (2015). Current pharmacological management of hypotensive syndromes in the elderly. Drugs Aging 32 (5), 337–348. 10.1007/s40266-015-0263-z 25948549

[B6] Alqenae F. A. Steinke D. Keers R. N. (2020). Prevalence and nature of medication errors and medication-related harm following discharge from hospital to community settings: A systematic review. Drug Saf. 43 (6), 517–537. 10.1007/s40264-020-00918-3 32125666 PMC7235049

[B7] Anrys P. Petit A. E. Thevelin S. Sallevelt B. Drenth C. Soiza R. L. (2021). An international consensus list of potentially clinically significant drug-drug interactions in older people. J. Am. Med. Dir. Assoc. 22 (10), 2121–2133.e24. 10.1016/j.jamda.2021.03.019 33901428

[B8] Bakris G. L. Fonseca V. Katholi R. E. McGill J. B. Messerli F. H. Phillips R. A. (2004). Metabolic effects of Carvedilol vs metoprolol in patients with type 2 diabetes mellitus and hypertension: A randomized controlled trial. JAMA 292 (18), 2227–2236. 10.1001/jama.292.18.2227 15536109

[B9] Bansilal S. Castellano J. M. Fuster V. (2015). Global burden of CVD: Focus on secondary prevention of cardiovascular disease. Int. J. Cardiol. 201 (1), S1–S7. 10.1016/S0167-5273(15)31026-3 26747389

[B10] Becker M. L. Kallewaard M. Caspers P. W. Visser L. E. Leufkens H. G. Stricker B. H. (2007). Hospitalisations and emergency department visits due to drug-drug interactions: A literature review. Pharmacoepidemiol. Drug Saf. 16 (6), 641–651. 10.1002/pds.1351 17154346

[B11] Beinse G. Reitter D. Segaux L. Carvahlo-Verlinde M. Rousseau B. Tournigand C. (2020). Potential drug-drug interactions and risk of unplanned hospitalization in older patients with cancer: A survey of the prospective elcapa (ELderly CAncer PAtients) cohort. J. Geriatr. Oncol. 11 (4), 586–592. 10.1016/j.jgo.2019.07.023 31445850

[B12] Bellosta S. Corsini A. (2018). Statin drug interactions and related adverse reactions: An update. Expert Opin. Drug Saf. 17 (1), 25–37. 10.1080/14740338.2018.1394455 29058944

[B13] Bertoccini L. Baroni M. G. (2021). GLP-1 receptor agonists and SGLT2 inhibitors for the treatment of type 2 diabetes: New insights and opportunities for cardiovascular protection. Adv. Exp. Med. Biol. 1307, 193–212. 10.1007/5584_2020_494 32034729

[B14] Celebi R. Uyar H. Yasar E. Gumus O. Dikenelli O. Dumontier M. (2019). Evaluation of knowledge graph embedding approaches for drug-drug interaction prediction in realistic settings. BMC Bioinforma. 20 (1), 726. 10.1186/s12859-019-3284-5 PMC6921491 31852427

[B15] Dagdelen M. S. Gulen D. Ceylan I. Girgin N. K. (2021). Evaluation of potential drug-drug interactions in intensive care unit. Eur. Rev. Med. Pharmacol. Sci. 25 (18), 5801–5806. 10.26355/eurrev_202109_26798 34604971

[B16] Davies E. A. O'Mahony M. S. (2015). Adverse drug reactions in special populations - the elderly. Br. J. Clin. Pharmacol. 80 (4), 796–807. 10.1111/bcp.12596 25619317 PMC4594722

[B18] Ersoy 0. Ersoy P. (2021). Effects of new drug interaction index on drug adherence in older patients with hypertension. Turk Kardiyol. Dern. Ars. 49 (7), 545–552. 10.5543/tkda.2021.21869 34623297

[B19] Ferrari R. Pavasini R. Censi S. Squeri A. Rosano G. (2021). The new ESC guidelines for the diagnosis and management of chronic coronary syndromes: The good and the not so good. Curr. Probl. Cardiol. 46 (3), 100554. 10.1016/j.cpcardiol.2020.100554 32173068

[B20] Fettah H. Moutaouakkil Y. Sefrioui M. R. Moukafih B. Bousliman Y. Bennana A. (2018). Detection and analysis of drug-drug interactions among hospitalized cardiac patients in the mohammed V military teaching hospital in Morocco. Pan Afr. Med. J. 29, 225. 10.11604/pamj.2018.29.225.14169 30100979 PMC6080962

[B21] Forman D. E. Maurer M. S. Boyd C. Brindis R. Salive M. E. Horne F. M. (2018). Multimorbidity in older adults with cardiovascular disease. J. Am. Coll. Cardiol. 71 (19), 2149–2161. 10.1016/j.jacc.2018.03.022 29747836 PMC6028235

[B22] Franz C. C. Egger S. Born C. Rätz B. A. Krähenbühl S. (2012). Potential drug-drug interactions and adverse drug reactions in patients with liver cirrhosis. Eur. J. Clin. Pharmacol. 68 (2), 179–188. 10.1007/s00228-011-1105-5 21842337

[B23] Gallo P. De Vincentis A. Pedone C. Nobili A. Tettamanti M. Gentilucci U. V. (2019). Drug-drug interactions involving CYP3A4 and P-glycoprotein in hospitalized elderly patients. Eur. J. Intern. Med. 65, 51–57. 10.1016/j.ejim.2019.05.002 31084979

[B24] Gatenby J. Blomqvist M. Burke R. Ritchie A. Gibson K. Patanwala A. E. (2020). Adverse events targeted by drug-drug interaction alerts in hospitalized patients. Int. J. Med. Inf. 143, 104266. 10.1016/j.ijmedinf.2020.104266 32961505

[B25] Gelchu T. Abdela J. (2019). Drug therapy problems among patients with cardiovascular disease admitted to the medical ward and had a follow-up at the ambulatory clinic of hiwot fana specialized university hospital: The case of a tertiary hospital in eastern Ethiopia. SAGE Open Med. 7, 2050312119860401. 10.1177/2050312119860401 31367379 PMC6643177

[B26] Gómez-Huelgas R. González D. Abadias M. Puig J. Ena J. (2020). Prescription patterns of antihyperglycemic drugs in elderly patients in Spain: A national cross-sectional study. Rev. Clin. Esp. 220 (3), 155–161. 10.1016/j.rce.2019.05.011 31326081

[B27] Good C. W. Steinhubl S. R. Brennan D. M. Lincoff A. M. Topol E. J. Berger P. B. (2012). Is there a clinically significant interaction between calcium channel antagonists and clopidogrel?: Results from the clopidogrel for the reduction of events during observation (CREDO) trial. Circ. Cardiovasc. Interv. 5 (1), 77–81. 10.1161/CIRCINTERVENTIONS.111.963405 22319066 PMC11610745

[B28] Grandchamp S. Blanc A. L. Roussel M. Tagan D. Sautebin A. Dobrinas-Bonazzi M. (2022). Pharmaceutical interventions on hospital discharge prescriptions: Prospective observational study highlighting challenges for community pharmacists. Drugs Real World Outcomes 9 (2), 253–261. 10.1007/s40801-021-00288-x 34971408 PMC9114175

[B29] Gremmel T. Durstberger M. Eichelberger B. Koppensteiner R. Panzer S. (2015). Calcium-channel blockers attenuate the antiplatelet effect of clopidogrel. Cardiovasc. Ther. 33 (5), 264–269. 10.1111/1755-5922.12138 26014752

[B30] Hessami A. Shamshirian A. Heydari K. Pourali F. Alizadeh-Navaei R. Moosazadeh M. (2021). Cardiovascular diseases burden in COVID-19: Systematic review and meta-analysis. Am. J. Emerg. Med. 46, 382–391. 10.1016/j.ajem.2020.10.022 33268238 PMC7561581

[B31] Hines L. E. Murphy J. E. (2011). Potentially harmful drug-drug interactions in the elderly: A review. Am. J. Geriatr. Pharmacother. 9 (6), 364–377. 10.1016/j.amjopharm.2011.10.004 22078863

[B32] Horn J. Ueng S. (2019). The effect of patient-specific drug-drug interaction alerting on the frequency of alerts: A pilot study. Ann. Pharmacother. 53 (11), 1087–1092. 10.1177/1060028019863419 31296026

[B33] Horodinschi R. N. Stanescu A. Bratu O. G. Pantea S. A. Radavoi D. G. Diaconu C. C. (2019). Treatment with statins in elderly patients. Med. Kaunas. 55 (11), 721. 10.3390/medicina55110721 PMC6915405 31671689

[B34] Jain V. Patel R. K. Kapadia Z. Galiveeti S. Banerji M. Hope L. (2017). Drugs and hyperglycemia: A practical guide. Maturitas 104, 80–83. 10.1016/j.maturitas.2017.08.006 28923179

[B35] Kheshti R. Aalipour M. Namazi S. (2016). A comparison of five common drug-drug interaction software programs regarding accuracy and comprehensiveness. J. Res. Pharm. Pract. 5 (4), 257–263. 10.4103/2279-042X.192461 27843962 PMC5084483

[B36] Kim J. Park S. Kim H. Je N. K. (2019). National trends in metformin-based combination therapy of oral hypoglycaemic agents for type 2 diabetes mellitus. Eur. J. Clin. Pharmacol. 75 (12), 1723–1730. 10.1007/s00228-019-02751-9 31475315

[B37] Knuuti J. Wijns W. Saraste A. Capodanno D. Barbato E. Funck-Brentano C. (2020). 2019 ESC guidelines for the diagnosis and management of chronic coronary syndromes. Eur. Heart J. 41 (3), 407–477. 10.1093/eurheartj/ehz425 31504439

[B38] Kovačević M. Vezmar K. S. Radovanović S. Stevanović P. Miljković B. (2020). Potential drug-drug interactions associated with clinical and laboratory findings at hospital admission. Int. J. Clin. Pharm. 42 (1), 150–157. 10.1007/s11096-019-00951-y 31865593

[B39] Lea M. Mowe M. Mathiesen L. Kvernrød K. Skovlund E. Molden E. (2019). Prevalence and risk factors of drug-related hospitalizations in multimorbid patients admitted to an internal medicine ward. PLoS One 14 (7), e0220071. 10.1371/journal.pone.0220071 31329634 PMC6645516

[B40] Limandri B. J. (2020). Adverse events, drug interactions, and treatment adherence. J. Psychosoc. Nurs. Ment. Health Serv. 58 (2), 9–13. 10.3928/02793695-20200117-02 32003860

[B41] Lineberry E. Rozycki E. Jordan T. A. Mellett J. North A. M. (2021). Implementation of pharmacist targeted discharge prescription review in an emergency department. Am. J. Emerg. Med. 48, 288–294. 10.1016/j.ajem.2021.04.054 34023809

[B42] Lopes R. D. Hong H. Harskamp R. E. Bhatt D. L. Mehran R. Cannon C. P. (2020). Optimal antithrombotic regimens for patients with atrial fibrillation undergoing percutaneous coronary intervention: An updated network meta-analysis. JAMA Cardiol. 5 (5), 582–589. 10.1001/jamacardio.2019.6175 32101251 PMC7240352

[B43] Magro L. Moretti U. Leone R. (2012). Epidemiology and characteristics of adverse drug reactions caused by drug-drug interactions. Expert Opin. Drug Saf. 11 (1), 83–94. 10.1517/14740338.2012.631910 22022824

[B44] Mahboobipour A. A. Baniasadi S. (2021). Clinically important drug-drug interactions in patients admitted to hospital with COVID-19: Drug pairs, risk factors, and management. Drug Metabol. Drug Interact. 36 (1), 9–16. 10.1515/dmpt-2020-0145 33580642

[B45] Malki M. A. Pearson E. R. (2020). Drug-drug-gene interactions and adverse drug reactions. Pharmacogenomics J. 20 (3), 355–366. 10.1038/s41397-019-0122-0 31792369 PMC7253354

[B46] Michniewicz E. Mlodawska E. Lopatowska P. Tomaszuk-Kazberuk A. Malyszko J. (2018). Patients with atrial fibrillation and coronary artery disease - double trouble. Adv. Med. Sci. 63 (1), 30–35. 10.1016/j.advms.2017.06.005 28818746

[B47] Moradi O. Karimzadeh I. Davani-Davari D. Shafiekhani M. Sagheb M. M. Raees-Jalali G. A. (2020). Drug-drug interactions among kidney transplant recipients in the outpatient setting. Int. J. Organ Transpl. Med. 11 (4), 185–195. PMC7726842 33335699

[B48] Moura C. S. Acurcio F. A. Belo N. O. (2009). Drug-drug interactions associated with length of stay and cost of hospitalization. J. Pharm. Pharm. Sci. 12 (3), 266–272. 10.18433/j35c7z 20067703

[B49] Nazarzadeh M. Bidel Z. Canoy D. Copland E. Wamil M. Majert J. (2021). Blood pressure lowering and risk of new-onset type 2 diabetes: An individual participant data meta-analysis. Lancet 398 (10313), 1803–1810. 10.1016/S0140-6736(21)01920-6 34774144 PMC8585669

[B50] Nightingale G. Pizzi L. T. Barlow A. Barlow B. Jacisin T. McGuire M. (2018). The prevalence of major drug-drug interactions in older adults with cancer and the role of clinical decision support software. J. Geriatr. Oncol. 9 (5), 526–533. 10.1016/j.jgo.2018.02.001 29510896

[B51] Ning C. Su S. Li J. Kong D. Cai H. Qin Z. (2021). Evaluation of a clinically relevant drug-drug interaction between rosuvastatin and clopidogrel and the risk of hepatotoxicity. Front. Pharmacol. 12, 715577. 10.3389/fphar.2021.715577 34646133 PMC8504577

[B52] Oliveros E. Patel H. Kyung S. Fugar S. Goldberg A. Madan N. (2020). Hypertension in older adults: Assessment, management, and challenges. Clin. Cardiol. 43 (2), 99–107. 10.1002/clc.23303 31825114 PMC7021657

[B53] Pejčić A. V. Janković S. M. Davidović G. (2019). Drug-drug interactions in patients with acute coronary syndrome across phases of treatment. Intern. Emerg. Med. 14 (3), 411–422. 10.1007/s11739-018-1994-8 30483990

[B54] Pinheiro L. F. França C. N. Izar M. C. Barbosa S. P. Bianco H. T. Kasmas S. H. (2012). Pharmacokinetic interactions between clopidogrel and rosuvastatin: Effects on vascular protection in subjects with coronary heart disease. Int. J. Cardiol. 158 (1), 125–129. 10.1016/j.ijcard.2012.04.051 22569318

[B55] Plácido A. I. Herdeiro M. T. Morgado M. Figueiras A. Roque F. (2020). Drug-related problems in home-dwelling older adults: A systematic review. Clin. Ther. 42 (4), 559–572.e14. 10.1016/j.clinthera.2020.02.005 32147147

[B56] Prince M. J. Wu F. Guo Y. Gutierrez R. L. O'Donnell M. Sullivan R. (2015). The burden of disease in older people and implications for health policy and practice. Lancet 385 (9967), 549–562. 10.1016/S0140-6736(14)61347-7 25468153

[B57] Ramsdale E. Mohamed M. Yu V. Otto E. Juba K. Awad H. (2022). Polypharmacy, potentially inappropriate medications, and drug-drug interactions in vulnerable older adults with advanced cancer initiating cancer treatment. Oncologist 27, e580–e588. 10.1093/oncolo/oyac053 35348764 PMC9255971

[B58] Ren W. Liu Y. Zhang J. Fang Z. Fang H. Gong Y. (2020). Prevalence of potential drug-drug interactions in outpatients of a general hospital in China: A retrospective investigation. Int. J. Clin. Pharm. 42 (4), 1190–1196. 10.1007/s11096-020-01068-3 32488437 PMC7476976

[B59] Riu-Viladoms G. Carcelero S. M. E. Martín-Conde M. T. Creus N. (2019). Drug interactions with oral antineoplastic drugs: The role of the pharmacist. Eur. J. Cancer Care 28 (1), e12944. 10.1111/ecc.12944 30324634

[B60] Romero-Farina G. Aguadé-Bruix S. (2021). Planning the follow-up of patients with stable chronic coronary artery disease. Diagn. (Basel) 11 (10), 1762. 10.3390/diagnostics11101762 PMC8535144 34679460

[B61] Ruangritchankul S. Peel N. M. Hanjani L. S. Gray L. C. (2020). Drug related problems in older adults living with dementia. PLoS One 15 (7), e0236830. 10.1371/journal.pone.0236830 32735592 PMC7394402

[B62] Seid E. Engidawork E. Alebachew M. Mekonnen D. Berha A. B. (2020). Evaluation of drug therapy problems, medication adherence and treatment satisfaction among heart failure patients on follow-up at a tertiary care hospital in Ethiopia. PLoS One 15 (8), e0237781. 10.1371/journal.pone.0237781 32857798 PMC7454938

[B63] Shehab N. Lovegrove M. C. Geller A. I. Rose K. O. Weidle N. J. Budnitz D. S. (2016). US emergency department visits for outpatient Adverse drug events, 2013-2014. JAMA 316 (20), 2115–2125. 10.1001/jama.2016.16201 27893129 PMC6490178

[B64] Shetty V. Chowta M. N. Chowta K. N. Shenoy A. Kamath A. Kamath P. (2018). Evaluation of potential drug-drug interactions with medications prescribed to geriatric patients in a tertiary care hospital. J. Aging Res. 2018, 5728957. 10.1155/2018/5728957 30402286 PMC6198551

[B65] Silber S. (2019). ESC guidelines 2019 on chronic coronary syndrome (CCS, previously "stable coronary artery disease"): What is new? What is particularly important? Herz 44 (8), 676–683. 10.1007/s00059-019-04862-6 31712870

[B66] Silva C. Ramalho C. Luz I. Monteiro J. Fresco P. (2015). Drug-related problems in institutionalized, polymedicated elderly patients: Opportunities for pharmacist intervention. Int. J. Clin. Pharm. 37 (2), 327–334. 10.1007/s11096-014-0063-2 25637404

[B67] Soejono C. H. Rizka A. (2021). Polypharmacy and drug use pattern among Indonesian elderly patients visiting emergency unit. Acta Med. Indones. 53 (1), 60–76. 33818408

[B68] Sönnerstam E. Sjölander M. Lövheim H. Gustafsson M. (2018). Clinically relevant drug-drug interactions among elderly people with dementia. Eur. J. Clin. Pharmacol. 74 (10), 1351–1360. 10.1007/s00228-018-2514-5 29967921 PMC6132551

[B69] Tavazzi L. Maggioni A. P. Marchioli R. Barlera S. Franzosi M. G. Latini R. (2008). Effect of rosuvastatin in patients with chronic heart failure (the GISSI-HF trial): A randomised, double-blind, placebo-controlled trial. Lancet 372 (9645), 1231–1239. 10.1016/S0140-6736(08)61240-4 18757089

[B70] Thomsen L. A. Winterstein A. G. Søndergaard B. Haugbølle L. S. Melander A. (2007). Systematic review of the incidence and characteristics of preventable Adverse drug events in ambulatory care. Ann. Pharmacother. 41 (9), 1411–1426. 10.1345/aph.1H658 17666582

[B71] Tsige A. W. Yikna B. B. Altaye B. M. (2021). Drug-related problems among ambulatory heart failure patients on follow-up at debre berhan comprehensive specialized hospital, Ethiopia. Ther. Clin. Risk Manag. 17, 1165–1175. 10.2147/TCRM.S337256 34785901 PMC8591109

[B72] Uijtendaal E. V. van Harssel L. L. Hugenholtz G. W. Kuck E. M. Zwart-van R. J. Cremer O. L. (2014). Analysis of potential drug-drug interactions in medical intensive care unit patients. Pharmacotherapy 34 (3), 213–219. 10.1002/phar.1395 24390929

[B73] Vecchia S. Orlandi E. Confalonieri C. Damonti E. Riva A. Sartori A. (2018). Prevalence study on potential drug-drug interaction in cancer patients in piacenza hospital's onco-haematology department. J. Oncol. Pharm. Pract. 24 (7), 490–493. 10.1177/1078155217717324 28714379

[B74] Veloso R. Figueredo T. P. Barroso S. Nascimento M. Reis A. (2019). Factors associated with drug interactions in elderly hospitalized in high complexity hospital. Cien. Saude Colet. 24 (1), 17–26. 10.1590/1413-81232018241.32602016 30698236

[B75] Wang Z. Y. Chen M. Zhu L. L. Yu L. S. Zeng S. Xiang M. X. (2015). Pharmacokinetic drug interactions with clopidogrel: Updated review and risk management in combination therapy. Ther. Clin. Risk Manag. 11, 449–467. 10.2147/TCRM.S80437 25848291 PMC4373598

[B76] Yasuda S. Miyamoto Y. Ogawa H. (2018). Current status of cardiovascular medicine in the aging society of Japan. Circulation 138 (10), 965–967. 10.1161/CIRCULATIONAHA.118.035858 30354536

[B77] Yoon S. J. Kim J. S. Jung J. G. Ahn S. K. Song Y. S. Bae N. K. (2018). Factors associated with potentially harmful drug-drug interactions in older Korean people: A population-based study. Geriatr. Gerontol. Int. 18 (9), 1378–1382. 10.1111/ggi.13495 30094910

[B78] Zahmatkeshan N. Khademian Z. Zarshenas L. Rakhshan M. (2021). Experience of adherence to treatment among patients with coronary artery disease during the COVID-19 pandemic: A qualitative study. Health promot. Perspect. 11 (4), 467–475. 10.34172/hpp.2021.59 35079592 PMC8767076

[B79] Zhao D. Liu J. Wang M. Zhang X. Zhou M. (2019). Epidemiology of cardiovascular disease in China: Current features and implications. Nat. Rev. Cardiol. 16 (4), 203–212. 10.1038/s41569-018-0119-4 30467329

[B80] Zhao M. Song J. X. Zheng F. F. Huang L. Feng Y. F. (2021). Potentially inappropriate medication and associated factors among older patients with chronic coronary syndrome at hospital discharge in beijing, China. Clin. Interv. Aging 16, 1047–1056. 10.2147/CIA.S305006 34135577 PMC8200161

