# Supplementary material for: Potential drug-drug interactions in drug therapy for older adults with chronic coronary syndrome at hospital discharge: A real-world study
Source: Front Pharmacol. 2022 Aug 24;13:946415. doi: 10.3389/fphar.2022.946415 (PMC9449411; doi:10.3389/fphar.2022.946415)
Supplement: Supplementary file 1 [file Table1.docx]

**Supplementary Table 1.** Drug classes implicated in pDDIs at discharge

| **ATC groups** | **C** | **D and X** | **n (%)** |
| --- | --- | --- | --- |
| C cardiovascular system | 806 | 11 | 817 (47.3) |
| ACE inhibitors | 50 | 0 |  |
| angiotensin Ⅱ inhibitors | 51 | 0 |  |
| β blocking agents | 126 | 0 |  |
| calcium channel blockers | 179 | 3 |  |
| diuretics | 176 | 2 |  |
| doxazosin | 2 | 0 |  |
| peripheral vasodilators | 19 | 0 |  |
| antiarrhythmics, class Ⅰ and Ⅲ | 45 | 2 |  |
| digoxin | 14 | 0 |  |
| lipid modifying agents | 144 | 4 |  |
| A alimentary tract and metabolism | 291 | 141 | 432 (25.0) |
| drugs used in diabetes | 266 | 130 |  |
| proton pump inhibitors | 9 | 5 |  |
| propulsives | 0 | 1 |  |
| mineral supplements | 13 | 5 |  |
| vitamins | 3 | 0 |  |
| B blood and blood forming organs | 340 | 72 | 412 (23.8) |
| vitamin K antagonist | 6 | 3 |  |
| antiplatelets | 331 | 41 |  |
| anticoagulants | 3 | 26 |  |
| iron preparations | 0 | 2 |  |
| N nervous system | 31 | 6 | 37 (2.1) |
| antiepileptics | 4 | 4 |  |
| psycholeptics | 6 | 1 |  |
| antidepressants | 15 | 0 |  |
| antivertigo preparations | 2 | 0 |  |
| anti-Parkinson drugs | 4 | 1 |  |
| H systemic hormonal preparations | 6 | 1 | 7 (0.4) |
| thyroid preparations | 3 | 1 |  |
| corticosteroids for systemic use | 3 | 0 |  |
| J antiinfectives for systemic use | 7 | 2 | 9 (0.5) |
| quinolone antibacterials | 6 | 1 |  |
| triazole derivatives | 1 | 1 |  |
| L antineoplastic and immunomodulating agents | 3 | 1 | 4 (0.2) |
| immunosuppressants | 2 | 1 |  |
| antineoplastic agents | 1 | 0 |  |
| M musculo-skeletal system | 9 | 0 | 9 (0.5) |
| antigout preparations | 7 | 0 |  |
| alendronic acid | 2 | 0 |  |
| G genitourinary system and sex hormones | 1 | 0 | 1(0.1) |
| sex hormones and modulators of the genital system | 1 | 0 |  |
| total | 1494 | 234 | 1728 (100.0) |

**Abbreviations:** ACE, angiotensin converting enzyme; ATC, anatomical therapeutic chemical group; pDDIs, potential drug-drug interactions.
